# Empowering Older Adults Through Digital Training: Facilitating Demand-Responsive Transport Adoption

**DOI:** 10.1093/geroni/igaf122.1997

**Published:** 2025-12-31

**Authors:** Ern Chern Khor, Donghun Kang, Jiwon Park, Wan Hong, Jungwoo Song, Moon Choi

**Affiliations:** Korea Advanced Institute of Science & Technology, Daejeon, Republic of Korea; Korea Advanced Institute of Science & Technology, Daejeon, Republic of Korea; Korea Advanced Institute of Science & Technology, Daejeon, Republic of Korea; Korea Advanced Institute of Science & Technology, Daejeon, Republic of Korea; Korea Advanced Institute of Science & Technology, Daejeon, Republic of Korea; Korea Advanced Institute of Science & Technology, Daejeon, Republic of Korea

## Abstract

Demand-responsive transport (DRT) services effectively address mobility needs in rural and low-density areas where public transportation options are limited. In South Korea, Shucle, a DRT service, offers shared rides within designated zones, dynamically adjusting routes based on demand. However, as these services rely on smartphone apps, older adults—who often constitute the majority of residents in such areas—face challenges in accessing them due to limited digital literacy. This intervention study aimed to develop and evaluate a training program to enhance older adults’ ability to use the Shucle app. Participants (n = 57, aged 61–81) were categorized into three groups based on their digital literacy. The training followed a structured sequence: (a) pre-assessment, (b) step-by-step guidance, (c) hands-on practice with Naver Map (the most popular digital map) and the Shucle app, (d) real-ride experience, and (e) post-assessment. Given that digital map literacy is essential for DRT adoption, the training prioritized navigation skills. Initially, nearly half of the participants struggled with Naver Map; however, post-training, over 90% successfully used it. Once proficient in digital mapping, most participants adapted quickly to the Shucle app, confirming the foundational role of navigation skills in DRT usage. Notably, those with moderate digital literacy demonstrated the greatest improvement in app proficiency and attitudes toward DRT services. These findings highlight the importance of tailored interventions to support older adults with varying digital competencies, ensuring equitable access to emerging mobility solutions.

